# Using Two Dyes with the Same Fluorophore to Monitor Cellular Calcium Concentration in an Extended Range

**DOI:** 10.1371/journal.pone.0055778

**Published:** 2013-02-07

**Authors:** Lourdes Figueroa, Vyacheslav M. Shkryl, Lothar A. Blatter, Eduardo Ríos

**Affiliations:** 1 Section of Cellular Signaling, Department of Molecular Biophysics and Physiology, Rush University, Chicago, Illinois, United States of America; 2 Department of General Physiology of the Nervous System, Laboratory of Biophysics of Ion Channels, A.A. Bogomoletz Institute of Physiology, Kiev, Ukraine; University of Queensland, Australia

## Abstract

We extend the sensitivity of quantitative concentration imaging to an approximately 1000-fold range of concentrations by a method that uses two fluorescent dyes with the same fluorophore, having different affinity for the monitored species. While the formulation and illustration refer to a monitor of calcium concentration, the method is applicable to any species that binds to multiple indicators with the same spectral properties. The use of a common fluorophore has the virtue of leaving vast regions of the electromagnetic spectrum available for other applications. We provide the exact analytic expression relating measured fluorescence to [Ca^2+^] at equilibrium and an approximate analytic expression that does not require the equilibrium assumption. The sensitivity of the method is calculated numerically for two useful dye pairs. As illustrative application of the enhanced measurement, we use fluo-4 and fluo-4FF to image the calcium wave produced by a cardiac myocyte in response to a small artificial calcium spark.

## Introduction

Optical sensors of concentration of a chemical species typically yield a signal (a change in fluorescence, absorbance or another measurable property) that grows with the concentration of the indicator bound to the monitored species. Therefore, the signals are limited at low concentrations by the sensitivity and at high concentration by the saturation of the sensor.

Research in cell physiology often calls for dynamic measurement in ranges wider than can be handled by a single monitor. Specifically, we found such need while exploring the response of cardiac and skeletal muscle cells to imposed increases in free [Ca^2+^]. The procedure was to apply as stimuli synthetic local increases in [Ca^2+^] (here called “artificial Ca^2+^ sparks”) [1], while monitoring the cellular response, expected to consist in release of Ca^2+^ from cellular stores. The applied artificial sparks could be varied in a wide range, from nM to tens of µM, while the cellular responses, the subject of the investigation, were also expected to vary widely. Many other situations can be envisioned that require the ability to monitor precisely the concentration of Ca^2+^ or other species over several orders of magnitude.

To achieve precision within an extended Ca^2+^ concentration range we implemented an approach using two fluorescent dyes with different affinities and a common fluorophore. The advantage of this approach is that it leaves other spectral regions free for additional fluorescence measurements, or other photoconversion applications. In the following we derive the equations necessary to interpret fluorescence images obtained with pairs of dyes and then provide an example use of the technique.

## Results and Discussion

### Theory

While the method was derived for and will be illustrated with Ca^2+^ sensors, the equations and procedures are independent of the nature of the monitored species. In our laboratory we have used Ca^2+^-sensitive dyes with the fluorescein fluorophore: fluo-4, with *K*_D_ = 0.45 µM (determined in our lab), fluo-4FF, with *K*_D_ = 9.7 µM and fluo-5N, with *K*_D_ = 90 µM (provided by manufacturers).

For the derivation we assume that the aqueous solution (typically equilibrated with the cytosol) contains a Ca^2+^ sensor D of dissociation constant *K*_D_ at total concentration *D*_T_, and a sensor E of dissociation constant *K*_E_ at concentration *E*_T_. At steady free concentration [Ca^2+^] the following equations apply:
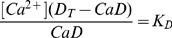
(1)

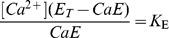
(2)And the fluorescence *F* is
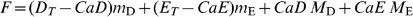
(3)where *CaD* and *CaE* are concentrations, and m_j_ and *M*_j_ represent the molar fluorescence of dye j in its free and Ca^2+^-bound forms.

*F* is a single-valued, monotonically increasing function of [Ca^2+^]. An explicit representation of it can be derived from Eqs. 1–3. This function can be inverted, to derive the free [Ca^2+^] from *F*, as follows
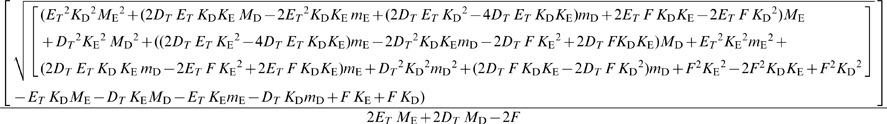
(4)(Analytical expressions for the concentrations *CaD* and *CaE* can also be derived from equations 1–3 and are available to the reader upon request).

**Figure 1:**
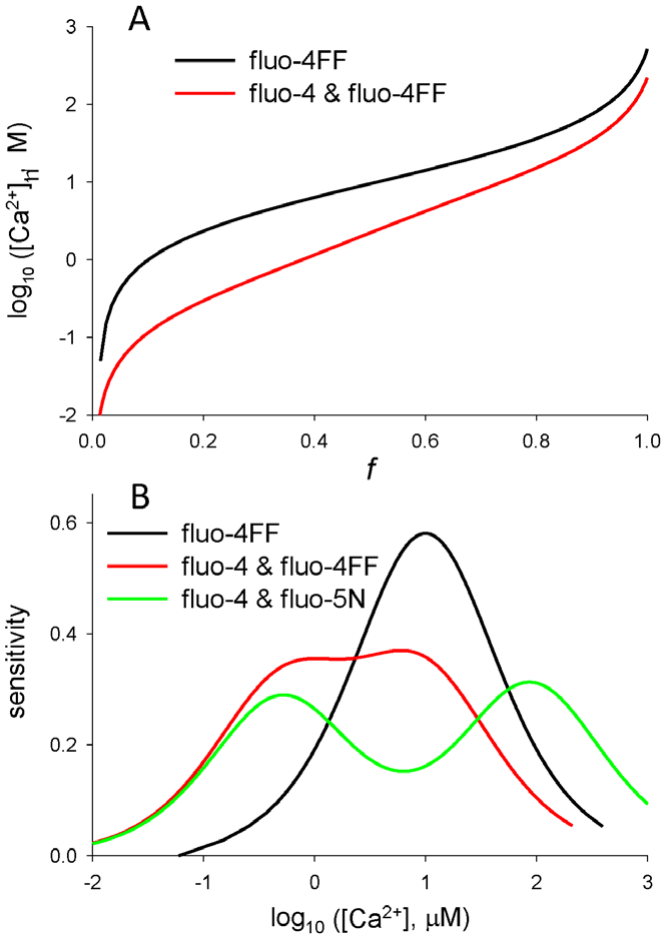
Combining the working ranges of Ca^2+^-sensitive dyes with the fluorescein fluorophore. *A*, the function relating steady free [Ca^2+^] to normalized fluorescence, for a single dye (black, equation 5) or two dyes (red, equation 4). *B*, the sensitivity, defined by equation 6, of the single dye (black), or pairs of dyes (red and green).

Fig. 1A plots, in red, log_10_ [Ca^2+^] vs. *f*, which is *F* normalized to its maximum (*D*_T_ *M*_D_+*E*_T_ *M*_E_), for fluo-4 and fluo-4FF used at equal concentrations. For comparison the same function for fluo-4FF alone is plotted in black. The dependence is in this case
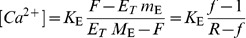
(5)where *R* is the dynamic range, *M*_E_/*m*_E_, of dye E.

The purpose of using two dyes is to extend the range of concentrations that can be measured with high sensitivity. The sensitivity of the dual dye method is compared with that of a single dye in Fig. 1B. The plot in black represents the sensitivity of the single dye, defined as
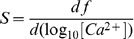
(6)namely, the slope of the corresponding function in Fig. 1A. The sensitivity, defined logarithmically, peaks at a concentration equal to the dissociation constant of the monitoring reaction. If the change in [Ca^2+^], rather than its log, is used in the definition, *S* will be maximal at [Ca^2+^] = 0 and decrease monotonically with increasing dye occupancy. In red is the sensitivity, defined again by function (6), for the two-dye case of fluo-4 and fluo-4FF. Given that the dissociation constants are 0.45 and 9.7 µM, the range of high sensitivity is extended to greater than two orders of magnitude. For an additional comparison we plotted in green trace the sensitivity attained with equal concentrations of fluo-4 and the low affinity dye fluo-5N.

In the representation of Fig. 1B the sensitivity of the two-dye methods is high in an extended range, but it is lower than the maximum attained with the single dye. This is strictly a matter of definition. If sensitivity is defined using the absolute fluorescence change, the two dyes will simply add their contributions.

The derivation above assumed equilibrium between both dyes and calcium. The assumption will not be valid when the goal is to monitor rapid changes with high affinity dyes. The equation that relates [Ca^2+^] to dye-Ca^2+^ concentrations without assuming equilibrium is
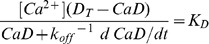
(7)and there is a corresponding equation for dye E.

We could not integrate analytically the partial differential system constituted by these kinetic equations and equation 3. We could however provide an approximate solution, with the reasonable assumption that the low affinity dye, E, is in equilibrium. The approximation therefore provides a kinetic correction for any lag in the reaction of dye D.

A first step is to recognize a relationship between the rates of change of *CaD* and fluorescence *F*.
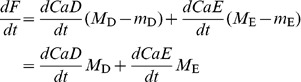
(8)where the simplification is valid for all non ratiometric dyes of high dynamic range, including the fluorescein-based ones in the example below.

An additional simplification should apply whenever the two dyes have very different affinities, namely, when the technique is used as intended. The rate of change of the concentration of the low affinity dye should be negligible in the concentration range detected by the high affinity dye, which is the only range where a kinetic correction is warranted. In this range
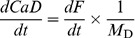
(9)which substituted in equation 7 yields
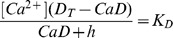
(10)
*h* represents the known term *M*_D_^−1^ *k*_off_^−1^ d*F*/d*t*.

The system of equations 2, 3 and 10 can be solved for [Ca^2+^], *CaD* and *CaE*. The expression for [Ca^2+^] is
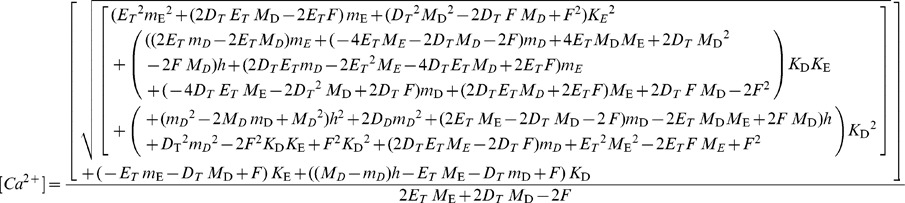
(11)It should be noted that the validity of this equation only extends to the range where equation 9 applies. Equation 11 does not apply when the low affinity dye contributes significantly to the rate of change of fluorescence. Conveniently, an increase in the contribution of the low affinity dye to the signal will in general also reduce the need for a kinetic correction.

Analytical expressions of the concentrations *CaD* and *CaE* with kinetic corrections, are also available to the interested reader.

### Experimental example

**Figure 2:**
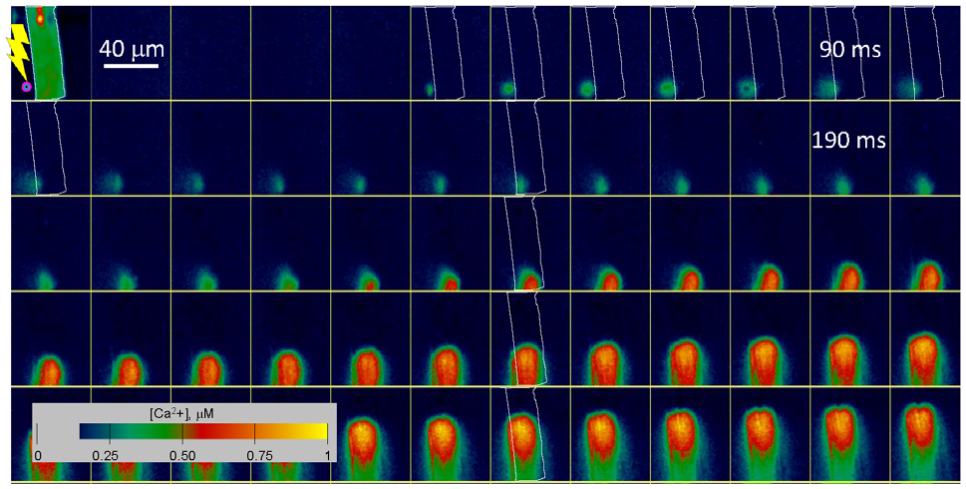
Two-dye images of stimulus and response in a cardiac cell. Images are of [Ca^2+^] derived by equation 4 from fluorescence of fluo-4 and fluo-4FF. The cell, an atrial cardiomyocyte, was enzymatically dissociated and its plasma membrane permeabilized by saponin. Frames, of which one of every 5 is shown, were acquired at 1.8 ms intervals. The first frame shows resting fluorescence and also the spot where IR light is applied, between the 5^th^ and 6^th^ frames, to un-cage Ca^2+^ from NP-EGTA and produce the artificial spark. Ca^2+^ in the spark reaches the membrane and elicits a propagated response. “Trigger [Ca^2+^]” is measured at the cell membrane in the frame marked “90 ms”. In selected frames, the cell contour traced on the first frame is reproduced in white. A slight offset of contour and Ca^2+^ transient in the last images reflects contractile movement.

As an example Fig. 2 shows cytosolic [Ca^2+^] in a rabbit atrial cardiomyocyte, derived from fluorescence of fluo-4 and fluo-4FF. The cell, whose plasmalemma was permeabilized with saponin, was immersed in an internal solution with known concentrations of both dyes and was stimulated by a small artificial Ca^2+^ spark. The spark was produced by two-photon lysis of the Ca^2+^ cage NP-EGTA, present at a concentration of 0.4 mM. Photolytic irradiation and image acquisition were carried out with a dual confocal scanner (LSM 5 DUO; Carl Zeiss, Oberkochen, Germany).

The resting fluorescence of the cell is represented in the first frame (in a highly expanded color scale). The artificial spark was produced outside the cell by brief irradiation at a diffraction-limited spot 3 µm away from the plasmalemma (target in first frame) with IR light pulsed for two-photon excitation. Images were acquired at one frame every 1.8 ms. The figure shows one every five images of this set (therefore the interval between the frames shown is 9 ms).

The cellular response, consisting in Ca^2+^ release from the sarcoplasmic reticulum, results in a propagating Ca^2+^ wave. The extended range of the two-dye technique allows for accurate, fast, nearly linear monitoring of the wave and precise imaging of the artificial spark. Note in particular the “trigger [Ca^2+^]” measured at the point where Ca^2+^ in the artificial spark reaches the cell membrane, at a time just before the first hint of a response (the frame at 90 ms). This trigger [Ca^2+^], 240 nM in the example, was just 50 nM above the resting [Ca^2+^] level. By systematically varying the intensity and duration of IR light, trigger [Ca^2+^] could be varied to find a minimum trigger, or “threshold [Ca^2+^]”, which could be defined with a precision of 10 nM. The ability to measure [Ca^2+^] in an extended range with a single fluorophore is being used to advantage in current applications, in which we add imaging of [Ca^2+^]_SR_ by SEER of fluorescence of mag-indo-1 [2], a ratiometric method that uses excitation and emission lights of lower wavelengths.

Some limitations in the applicability of the technique should be acknowledged. The use of two dyes of different affinity extends the measurable range of concentrations but cannot be construed as providing an extended range where linearity between signal and concentration applies. The linear range is limited to that of the low affinity dye. If the two affinities are very different there may be an additional approximately linear range, at concentrations where the high affinity dye is saturated.

The calculation of [Ca^2+^] with equation 4 is exact, unless it changes too rapidly compared with the dye reaction rates. The equation requires knowledge of both dye concentrations, which is accurate when cells are permeabilized, as in the example, but may constitute a problem in other cases.

In summary, the combination of two synthetic fluorescent sensors with the same fluorophore and different Ca^2+^ affinity allowed us to extend the range of concentrations that can be monitored with high precision. While the range of concentrations can be similarly extended using dyes of different spectra, the dual application of a single fluorophore preserves other regions of the spectrum for simultaneously monitoring other variables.

## Methods

### Ethics Statement

The experiment illustrated in Fig. 2 was carried out on single cardiomyocytes isolated from rabbit heart. The investigation conforms to the Guide for the Care and Use of Laboratory Animals of the National Institutes of Health. Procedures and protocols for animal handling and cell isolation were fully approved by the Institutional Animal Care and Use Committee of Rush University Medical Center (Animal Welfare Assurance number A-3120-01; specific protocol permit number 09-055). All efforts were made to minimize suffering.

### Preparation of cells and photorelease of caged Ca^2+^

Briefly, animals were anesthetized with thiopental sodium (50 mg kg−1, I.P.). After thoracotomy, hearts were excised, mounted on a Langendorff apparatus, and retrogradely perfused via the aorta with Liberase Blendzyme TH containing solution (37°C). Myocytes were used for experimentation within 1–6 hours after isolation. Myocytes adhered to the coverslip bottom of a custom chamber on the stage of a microscope, where its plasma membrane was permeabilized by saponin treatment (0.002% for 2 min). Photolytic irradiation and simultaneous image acquisition were carried out with a dual confocal scanner (LSM 5 DUO (Carl Zeiss, Oberkochen, Germany)) combining a slit scanner (LSM 5 *LIVE*) for rapid image acquisition with a conventional pinhole scanner (LSM 510) for irradiation. The spark was produced by two-photon lysis of the Ca^2+^ cage NP-EGTA, which was present in the experimental solution at a concentration of 0.4 mM and equilibrated with a free [Ca^2+^] of 190 nM. A diffraction-limited spot 3 µm away from the plasmalemma was irradiated for 1 ms with IR light of 710 nm pulsed for two-photon excitation. Images were acquired at one frame every 1.8 ms. [Ca^2+^] was derived from fluorescence according to equation 4. Parameters *D*_T_ and *E*_T_ were known in the applied solution. *D*_T_ and *E*_T_ inside the cell were calculated according to resting fluorescence, assuming that [Ca^2+^], *M*_D_, *m*_D_, *M*_E_ and *m*_E_ in cytosol were the same as in the solution, and that both dyes bound equally inside the cell. The molar fluorescence parameters were determined separately in solution for both dyes. Additional details of solution composition, perfusion, irradiation and image acquisition and processing were provided previously [3].
